# Examining a Novel Legacy Activity for Elders: Oral Histories as Produced Stories

**DOI:** 10.1089/pmr.2023.0032

**Published:** 2024-01-09

**Authors:** Tony H. Liu, Andrea Vernon-Cwik, Sandy Tun

**Affiliations:** ^1^ Pritzker School of Medicine, University of Chicago, Chicago, Illinois, USA.; ^2^ UChicago Medicine, Chicago, Illinois, USA.; ^3^ Department of Medicine, University of Chicago, Chicago, Illinois, USA.

**Keywords:** African American, dignity therapy, geriatric, legacy activity, media, narrative medicine, oral history, palliative care

## Abstract

**Background:**

Many African American elders who participated in The Great Migration are in the latter years of their lives. One way to maintain their memories and those of elders at large is through legacy activities, projects that initiate a life review process resulting in a product surviving after an individual's death. However, literature on culturally attuned legacy activities as well as measurement of impact are limited.

**Objectives:**

This project sought to introduce a novel legacy activity for elders—the oral history as produced aural self-story—detailing its creation and examining its therapeutic efficacy.

**Design, Setting, and Subjects:**

Nine African American elders who experienced The Great Migration receiving care from an urban, geriatric clinic were recruited. Oral histories were conducted, produced into aural self-stories, and examined with follow-up interviews and a project evaluation survey. Qualitative analysis of the follow-up interview and a project evaluation survey were used to ascertain therapeutic outcomes.

**Results:**

*:* All participants recommended the project and found self-story listening meaningful or beneficial. Qualitative interviews produced 13 codes; the five most frequent were *reflection/contemplation* (*n* = 18), *sentimentality/positive affect* and *affirmation/enlightenment* (*n* = 10), as well as *empathy/gratitude* and *curiosity/intrigue/peculiarity* (*n* = 7).

**Conclusion:**

*:* Our project suggests that aural self-stories produced from oral histories enhance the current elder legacy activity landscape by facilitating meaning and existential affirmation, additionally leaving a product for subsequent generations. Future studies include comparison to existing legacy interventions and project examination in additional elder populations.

## Key Message

This proof-of-concept study details the creation and efficacy of a novel form of legacy activity—the produced aural self-story—for elders. Qualitative analysis and project evaluation surveys suggest existential affirmation and meaning making for participants, demonstrating new avenues for integrating narrative and palliative medicine.

## Introduction

Between 1910 and 1970, millions of African Americans migrated from communities in the Deep South to northern industrial hubs. One prominent destination of this Great Migration was Chicago, particularly the city's South Side, which is still ∼75% African American.^1,2^ Building off an earlier study interviewing African American South Side Chicago elders who experienced the Great Migration and have now entered the latter years of their lives—the patient population for this project—we sought to ascertain whether a novel form of legacy activity, oral histories as produced aural self-stories, would provide therapeutic benefits for elders.

The produced aural self-story integrates dimensions of Dignity Therapy, Narrative Therapy, and psychotherapeutic models while taking the form of a legacy activity, a project that begins a life review process and results in a product surviving after an individual's death such as scrapbooking, writing stories on paper, or recording videos.^3–5^

However, the produced self-story reflects philosopher Adriana Cavarero's notion that “identity is not innate but rather revealed through narratives of self told by others,” thus representing a particular opportunity to elucidate an elder participant's longitudinal story of self.^6^ This revelation is also relevant for seniors when understood from phenomenological and identity-oriented frameworks; not only are seniors navigating Erikson's developmental stage of generativity—defined by the continuation of will and existence through relationships and memory^7,8^—they are also seeking to affirm existential meaning given the approach of their latter years of life as noted by Victor Frankl.^9–11^

In addition, while resonating with an elder's stage of life, the produced aural self-story also honors cultural dimensions of narrative storytelling and its therapeutic potential.^12–15^ Despite growing research on legacy activities, literature on culturally attuned activities as well as impact measurements of Dignity Therapy are limited.^16^ Consequently, the produced aural self-story emerged as one avenue to fill these gaps and as a means to promote the development of epistemic trust between patient and provider through listening, sharing, and witnessing.^17^

## Methods

### Study design

This study consisted of two parts: (1) oral history interview, which was produced into an aural story; and (2) aural story listening with follow-up interview and survey questions. The study team consisted of S.T., a practicing physician double boarded in internal medicine and hospice and palliative medicine, T.L., a medical student, and A.V.C., a licensed clinical social worker.

One interview conducted in January 2022 by S.T. and A.V.C. was used to produce an aural self-story. T.L. conducted the remaining 17 interviews in June and July 2022 with either A.V.C. and/or S.T. present and supervising T.L. for each interview. This study was approved by the University of Chicago Institutional Research Board (IRB #21-1614).

### Study population

Inclusion criteria consisted of individuals 55 and older who provided consent, experienced the Great Migration or Great Migration stories related to the South Side of Chicago, and identified as African American. Individuals outside of this inclusion criteria were excluded. All participants were referred by attending geriatrics physicians at the UChicago Medicine South Shore Senior Center (SSSC).

These physicians deemed patients as having both the cognitive capacity to give informed consent and a functional status that would allow for participation without undue burden. This resulted in list of 21 potential participants created in the electronic medical record software, Epic. Eligible participants were then called, informed of all project details and research intent, and asked about their desire to participate.

Six individuals, five of whom were women, declined to be interviewed, and six individuals, all women, expressed initial interest yet did not respond back. Thus, nine participants were recruited with the majority being men. Participants were instructed that they could bring a family member or friend to the interview. Each participant was provided a $50 gift card for participating in the oral history and another $50 gift card for the follow-up interview and listening session. Table 1 depicts participant demographics.

**Table 1.** Participant Demographics

Participant characteristic (***n*** = 9)	***n***	%
Gender		
Male	6	67
Race/Ethnicity		
African American	9	100
Age range		
70–79	1	11
80–89	5	56
90–99	3	33
Highest level of education		
High school	1	11
Some college	5	56
Some graduate school	3	33
Self-reported health		
Poor	1	11
Fair	1	11
Good	7	78
Marital status		
Single	1	11
Married	2	22
Divorced	3	33
Separated	1	11
Widowed	2	22

### Oral history interview

30-to-60-minute, semi-structured oral history interviews were conducted in-person at the SSSC. Questions stemmed from a pre-existing legacy questionnaire with additions from Chochinov et al.^18^ and Allen et al.^3^ The traditional Dignity Therapy script was altered to accommodate format differences specific to aural story therapy and production.

This form of therapy helps participants deconstruct their experiences to reconstruct a sense of self and identity^19^; subsequently, questions are related to narrative and salient experiences in life, including transitions, self-discovery, and affirmations of identity.^20^ Five key categories defined the project's questionnaire: (1) introduction, (2) early life experiences, (3) broader life experiences (work, relationships, self-identified significant experiences), (4) Chicago, and (5) reflective (Appendix A1). The study team did not share the question script in advanced to maintain journalistic standards around production where pre-formed answers influence interview outcomes.^20^

However, each participant was informed that they could share whatever they wished and felt comfortable to share and also that each could bring significant items or media to the interview. To accommodate participant preference, each was also informed that the study team would conduct any additions or removals to the produced aural story desired by a participant.

### Aural story production

T.L. established the production procedure and produced all oral histories into aural stories. Before each interview, the following equipment was set up: microphone and stand, an audio interface, headphones, a webcam for video recording, associated cables, and a digital audio workstation (DAW). Transcripts were made from the website Trint. Next, T.L. conducted an editorial listen, detailing the larger themes and content of the interview.

Then, using the DAW ProTools, T.L. began formal production conducting editorial and general clean-up, which consisted of removing non-participant audio, interview sections not deemed editorially relevant, and rearranging sections to best fit the narrative arc of a patient's life. Music, provided and composed by the musician Gautam Srikishan, was added, and sound levels were altered before the final mixdown of the digital aural story.

These stories ranged from ∼15–30 minutes in length, and the following participant provided consent to share their produced aural story https://uchicago.box.com/s/wo2j5m74n3z13bskfe4dncckacl65aaq.^21^ Table 2 depicts the complete aural story production workflow.

**Table 2.** Production Process

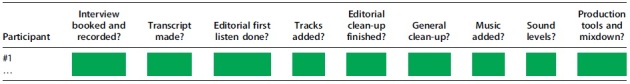

Green color indicates completion of step.

Production choices included the addition of non-semiotic elements such as music, oral history rearrangement, and decisions regarding what to remove or incorporate in the final story, ultimately reflecting subjectivity in production. However, key principles guided these decisions to offer rigor and standardization. Aside from including a specific song a participant named as significant, music never included human vocals or words to minimize the impact of semiotic meaning on a participant.

Next, music was necessary in the aural story medium as it facilitated movement through a participant's life narrative, a function proposed by theorist Jean Mitry for mediums constituted by time, including audio narration and film.^22^ Finally, music was included to distinguish a produced aural story from a simple audio recording; by including music, a produced piece signified to a participant that their life story was of value and worthy of production, an elevation of remembrance tied to the project's legacy activity goals.

**FIG. 1.**
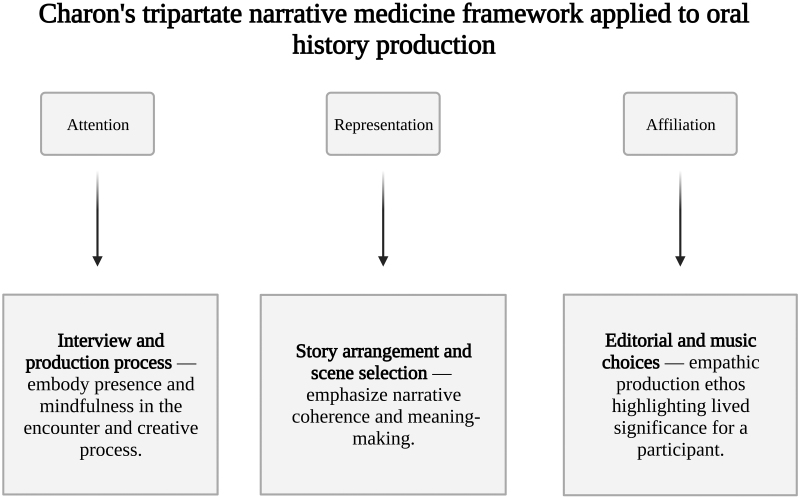
Rita Charon's narrative medicine framework applied to aural story production.

Additional production standardization included the application of Rita Charon's^23,24^ tripartite, narrative medicine framework of attention, representation, and affiliation (Fig. 1). Charon^24^ describes attention as attuning to a patient as “a recognizing vessel,” manifesting as presence and mindfulness during the interview, and representation as “taking a chaotic or formless experience and conferring form on it.”

We applied representation to oral history rearrangement, music, and story selection to emphasize the narrative arc of a patient's life facilitating awareness and meaning making.^25,26^ Affiliation reflects the contemplation of mortality and shared experience of illness between patient and caregiver. This provided an empathic production ethos for editorial and sonic choices predicated on highlighting lived significance for a participant.

Finally, ethical guidelines from journalistic standards^20^ guided production choices, including the non-alteration of a patient's semiotic meaning, where edits consisted of cutting out stumbles, pauses, and filler phrases such as “um.” To honor each participant's lived complexity, the research team made the full, unedited oral history available to each participant.

### Follow-up interview and surveys

Follow-up interviews were conducted in-person or through videoconferencing. This second interview consisted of a participant listening to their story followed by questions. The study team created questions with additions from the Hospice Foundation's *A Guide for Recalling and Retelling Your Life Story*^27,28^; questions fell under four categories: (1) the experience sharing one's story, (2) listening to the produced aural story, (3) investigating the produced piece, and (4) broader reflection. Interviews lasted 30–45 minutes. Finally, a project evaluation survey was administered after the listening session and follow-up interview.

### Analysis

Oral history and follow-up interview transcripts were coded for thematic analysis. This project used a semantic realist or essentialist perspective for textual analysis interpreting words as direct reflections of reality and thus meaning.^29^ Latent analysis refers to ideological exploration underpinning text that this project did not conduct.

However, if a response to a closed-ended question had additional follow-up by the participant, we proceeded to code the response. In addition, we conducted thematic analysis inductively using six phases^30^—(1) familiarization; (2) coding; (3) searching for themes; (4) reviewing themes; (5) defining and naming themes; concluding with (6) writing the report.

## Results

We identified 13 follow-up response codes: (1) reflection/contemplation, (2) sentimentality/general positive affect, (3) empathy/gratitude, (4) curiosity/intrigue/peculiarity, (5) incredulity/surprise, (6) numinosity, (7) affirmation/enlightenment/elucidation, (8) existentiality/legacy-related, (9) pride, (10) mundanity/neutrality, (11) nervousness/uncertainty, (12) discomfort/pain/sadness, and (13) regret. The top five codes were *reflection/contemplation* (*n* = 18), *sentimentality/positive affect* (*n* = 10), *affirmation/enlightenment* (*n* = 10), *empathy/gratitude* (*n* = 7), and *curiosity/intrigue/peculiarity* (*n* = 7).

On sharing one's history, participants described *gratitude*, *contemplation*—“It was different sharing it before you whom I didn't know. I wondered the truth of it”—and *sentimentality*, “In going within, there were times that I felt a little emotional, and I know that it was okay because I had to come from my truth, and that's important.” *Curiosity* was also a common refrain—“I've never had an experience like this before, to talk about myself and say off the top of my head”—as well as *nervousness*, “I really didn't know what was expected of me.”

Some participants reported a *contemplative state* after they finished telling their story*:* “I wasn't dismayed by it, but I just had the same wonderings of, ‘did it make sense?’ You don't have time often to construct what you're going to say. This just comes up automatically.”

However, participants more frequently described an *affirmed* or *enlightened* quality—“Me speaking it was like confirming, ‘This is part of my belief now. This is part of what I'm living.’” They also expressed feelings of *pride*, where one participant told the study team that they called their relatives about being invited to participate.

When asked what participants felt during or after the aural story listening experience, the primary response was *general positive affect*: “I felt very happy about remembering the people who had helped me.” *Gratitude* was also expressed—“I've talked about what I felt and what I said, but how you intuitively put that information together so cohesively and so beautifully.” In addition, *incredulity*—“At some point I thought, ‘Who's that? Who said that? Where did that come from?’”—was named.

While participants were told that produced stories would be unable to encapsulate all of a participant's life, some expressed *regret* at what they had chosen to share or not share. Responses varied regarding whether a participant would think of their lives any differently after listening, where one named that the experience helped them integrate life lessons (*reflection*)—“I guess what I did was supposed to be, and the thing that impressed me was today I learned from it. I learned from my mistakes. I learned to listen.”

Finally, participants were asked whether the experience listening to their produced story was valuable. All participants responded affirmatively. Some described the experience as *elucidating* and others as *contemplative*, “It was just good hearing it and understanding that if I were to write it, how I would expand it and fill in more gaps. But as a set of Cliff Notes, it wasn't bad. I had no trouble listening to it. I said that. I appreciate that. I knew that was me.” Unique to this question were responses about the project's *legacy value* for community or family—“Well, my grandkids, they're going to benefit from it you know?”

### Favorite sections and story changes

Music-related moments emerged as common favorite story sections. One participant reported their discussion of the guitarist Sister Rosetta Tharpe and her accompanying music as their favorite, and another participant described a seminal experience in their life during which Marvin Gaye's “What's Going On” was playing. Other favorite sections included scenes about family, jobs, and celebration as well as the emphasis of self-reported life lessons such as viewing one's mistakes as opportunities.

Regarding story sections participants disliked or would change, the majority reported nothing. When asked why, one participant said “because it's good to have experienced it. And by experience, I can look forward and move forward.” Two respondents said they wished they added more during the oral history, and one participant said that they would like to clarify that one section of the produced story was specifically addressed to the participant's children rather than being a general statement.

### Project evaluation survey

Each participant reported recommending the oral history and aural self-story listening experience and satisfaction with their project experience. When asked why, participants named there was benefit in reflection as well as in clarifying one's values. One participant reported medium satisfaction due to a dissatisfaction with self at not “being able to organize things…” regarding that participant's own telling of their life.

All participants reported that they found the experience listening to their aural story meaningful or beneficial. Participants stated that the story would assist their current lives—“I found it useful and beneficial because I still feel I have a lot of life ahead of me.”—and as an opportunity for reflection. Finally, participants also reported that being asked to participate was meaningful and that the produced stories reflected important social topics, “I think it shows benefit to the truth of how discrimination and stereotypes and racism occur.”

For interest in additional legacy activities, six expressed interest and three did not, where the non-interested individuals clarified that this project was sufficient or that they had already done legacy activities. Regarding additional comments, one participant named improved coaching about what a participant is allowed to share. Other comments reflected satisfaction and gratitude at the project—“When I say I appreciate you, I truly mean that…if you were just asking questions and doing a job of editing, you could have really screwed up something…but you didn't. You know, just superb.”

## Discussion

### On social desirability bias and patient participation

Given the intervention form, social desirability bias may have influenced participant responses in both interviews. While recognizing this influence, this project falls under a growing model in narrative medicine research that views patients as co-participants in the production of “shared narrative work between healthcare trainees and patients,”^31–33^

In this model, influence between researcher and participant is bidirectional. While we asked questions and produced the story, participants were given the ultimate authority on content with the power to add or remove sections from a produced piece. By providing participants this decisional power, participant honesty was incentivized as they could represent their story after the initial production with the research team accommodating their desires.

Thus, while social desirability bias did exist, this project's utilization of the patient-as-partner approach, what Charon calls “unifying seer and seen in the creation of the text,” suggests that social desirability bias was mediated as patient preferences and experiences were prioritized.^23,24,32^

### On intervention feasibility

Cercato et al. state that the success and adoption of digital, narrative medicine interventions are context-based, reflecting team participation and the particularities of a health care organization.^34,35^ Echoing this idea, while our senior clinic operates under a larger medical institution, it provides a community role in having established, longitudinal relationships with patients as well as coordinated primary care, including dedicated social workers with established rapport and trust.

Given this community and team-based approach, the barrier to entry to conduct interviews and produce oral histories was low, where these conditions may not exist in other health care environments for elders such as emergency settings. Having noted that produced aural stories are better served in community clinic settings, our project also suggests that additional entry barriers are not prohibitive.

While we utilized higher quality equipment for proof-of-concept purposes, free and open-source music and software exist to produce stories, and ubiquitous technologies such as smartphones can operate as quality microphones. Production work post-interview took two to three hours, less than the usual amount of time to produce an audio segment of similar length and style, where production time ultimately depends on a specific producer's background.^20^

While one must learn to use production software, rapid growth in podcasting from non-industry professionals and independent producers has expanded free and effective resources for pedagogy ranging from National Public Radio (NPR) training to extensive production guides from the public media organization Transom. Accordingly, under appropriate circumstances with clinic and team form, produced aural self-story interventions represent an opportunity to provide legacy development for elders to be shared with future generations.

## Conclusion

Our project suggests that aural self-stories produced from oral histories enhance the current legacy activity landscape for elders. Follow-up interview codes revealed that oral history sharing and listening to a produced aural self-story primarily facilitated *reflection/contemplation*, *sentimentality/positive affect*, *affirmation/enlightenment*, *empathy/gratitude*, and *curiosity/intrigue/peculiarity*, suggesting that produced aural self-stories fulfill the role of legacy activity and facilitate movement toward Erikson's stage of generativity.^7,8^

All participants also recommended the project and found the self-story listening experience meaningful or beneficial. Alongside participant recruitment, future project directions include randomized trials to compare efficacy with existing legacy interventions and an investigation of the intervention for other populations to expand generalizability.

Mattingly^26^ states that narrative produces significance and understanding by “[placing] temporality at the center of meaning,” a concept emphasized by our produced narratives and this participant's response: “Me speaking it was like confirming, ‘This is part of my belief now. This is part of what I'm living.’”

By expressing the qualitative sense of a life well-lived, the project experience aligns with Fonagy and Allison's^36^ notion of mentalizing in therapy, where feeling understood restores “a potential for learning about oneself and others in the world outside of therapy,” a way to honor and enrich the lives of the participating elders.

### Limitations

To maintain the safety of this vulnerable population, participants were referred by physicians; however, the referral process may have led to a non-response bias. Moreover, of those who responded, most were men suggesting an opportunity for improved sampling and recruitment. As described, this intervention may also suit specific health care environments such as primary care clinics more appropriately.

Terminally ill participants may also find this intervention taxing, where other legacy activities or an alternative version of the aural self-story process may function as an improved fit.^37^ Finally, given the scale and scope of the project, changes to each produced story requested after the study period were not feasible; although no participant requested a change after the study period, the study team imagines a potential for a digital archive in which comments and addendums can be made as a site of future investigation.

## Funding Information

This work was supported by The University of Chicago Pritzker School of Medicine, the Bucksbaum Institute for Clinical Excellence at UChicago Medicine, and a National Institute on Aging grant, no. 5T35AG029795-15.

## Abbreviations Used

DAW: digital audio workstation
NPR: National Public Radio
SSSC: South Shore Senior Center

## Appendix A1. Interview Guide, Project Evaluation Survey

Interview #1—Oral History Interview

1. Intro
  a. What's your name and where were you born?
  b. When were you born?
2. Early life
  a. When did you or your family arrive in Chicago?
  b. What is your earliest memory of Chicago?
  c. What hobbies or interests did you have as a younger person?
3. Broader life history
  a. Tell me a little about your life history; particularly the parts that you either remember most or think are the most important?
  b. What are the ideas, books, music, and poems that have most influenced your life?
  c. Were you married or did you have a life partner?
  *d. Feelings*
    *i.* When did you feel most alive?
    *ii.* What have been the most significant experiences in your life?
    *iii.* What are you most proud of in your life?
4. Work/roles
  *a.* What type of work did you do throughout your life?
  *b.* What are the most important roles you have played in life (family roles, vocational roles, community-service roles, etc.)? Why were they so important to you, and what do you think you accomplished in those roles?
5. On Chicago
  *a.* How has Chicago changed since you grew up?
  *b.* What is your favorite memory of Chicago?
6. Reflection
  *a.* What, if anything, would you have chosen to do differently in your life?
  *b.* How have your values changed during your life?
  *c.* What advice would you give to young people nowadays?
  *d.* How would you like your loved ones to remember you?
    *i.* Are there particular things that you feel still need to be said to your loved ones or things that you would want to take the time to say once again?
  *e.*  What are your hopes and dreams for your loved ones?
    *i.* Are there words or perhaps even instructions that you would like to offer your family to help prepare them for the future?
  *f.* What have you learned about life that you would want to pass along to others?
  *g.* In creating this permanent record, are there other things that you would like included?

Interview #2—After listening to produced aural story:

1. Story sharing
  a. What was it like telling your life story during the oral history?
  b. How did you feel after we finished?
2. Listening
  a. What feelings did you have listening to the recording of your story?
  b. How did you feel after you heard your story? Why do you think you felt that way?
  c. Were you surprised at any moment listening?
  d. How did recalling memories of family make the participant feel?
3. Production
  a. What was your favorite part of your story?
  b. Were there parts of the story you didn't like?
4. Reflection
  a. Do you think about your life any differently after listening to the story? If so, in what ways?
  b. How would you describe the experience of reflecting on your life?
  c. How would you describe the experience of listening to your life story as an audio story? Did you find the experience of valuable? If so, how?

Project Evaluation Survey:

1. Would the participant recommend the oral history and aural self-story listening experience to others?
2. Is the participant satisfied with the aural story and the experience of having an oral history conducted?
3. Did the participant find the experience of listening to one's aural story meaningful or beneficial?
4. Would the participant be interested in engaging in additional legacy activities (ex: journaling, scrapbook with photographs)?
5. What did the participant like about the oral history interview and the aural story?
6. What did the participant dislike or wish was changed about the oral history interview and the aural story?
7. What aspects of the oral history interview and the aural story worked well? What did not work as well?
8. Does the participant have any other thoughts, comments, or questions about the aural story?
